# Observation of Anesthetic Effect of Dexmedetomidine Combined With Intraspinal Anesthesia in Hip Arthroplasty and its Effect on Postoperative Delirium and Stress Response

**DOI:** 10.3389/fsurg.2022.928922

**Published:** 2022-07-01

**Authors:** Yading Shen, Chenyu Wang, Xiaoping Zhong, Yandan Wu, Xiaoxia He

**Affiliations:** ^1^ Department of Anesthesiology, Yiwu Central Hospital, Yiwu, China; ^2^ Department of Anesthesiology, Zhuji People's Hospital of Zhejiang Province, Zhuji, China

**Keywords:** hip replacement, dexmedetomidine, intraspinal anesthesia, anesthetic effects, stress response, delirium, immunologic function

## Abstract

**Objective:**

To observe the anesthetic effect of dexmedetomidine combined with spinal anesthesia in hip arthroplasty, and to analyze the effects of dexmedetomidine on postoperative stress response, incidence of delirium, immune function and inflammatory indicators.

**Methods:**

A total of 42 patients who underwent hip replacement in our hospital from March 2020 to June 2021 were selected as the research subjects and randomly divided into the control group and the observation group, 21 cases in each group. The control group was given intraspinal anesthesia, and the observation group was given dexmedetomidine on this basis. The onset time and maintenance time of sensory and motor nerve block were recorded. Stress response indexes [cortisol (Cor), blood glucose (Glu), adrenaline (E), noadrenaline (NE)], T lymphocyte subsets (CD3+, CD4+, CD8+, CD4+/CD8+), inflammatory indexes [tumor necrosis factor -*α* (TNF-α) and interleukin-6 (IL-6)] were detected before and after operation, and the incidence of postoperative delirium in both groups was recorded.

**Results:**

The onset time of sensory nerve block and motor block in the observation group were lower than those in the control group, and the retention time of sensory nerve block and motor nerve block were higher than those in the control group (*P* < 0.05). After surgery, the levels of Cor, Glu, E and NE in the observation group were lower than those in the control group (*P* < 0.05). After surgery, the incidence of postoperative delirium in the observation group (4.79%) was lower than that in the control group (28.57%) (*P* < 0.05). After surgery, the levels of CD3+, CD4+, CD8+, and CD4+/CD8+ in the observation group were higher than those in the control group (*P* < 0.05). After surgery, the levels of TNF-α and IL-6 in the observation group were lower than those in the control group (*P* < 0.05).

**Conclusion:**

The combined use of dexmedetomidine and intraspinal anesthesia has good anesthesia effect in hip joint replacement, which can greatly reduce the stress response of patients, reduce the incidence of postoperative delirium, and effectively restore the immune function of patients, reduce the level of inflammatory response, and has high clinical application value.

## Introduction

Hip is the area where the trunk and legs are connected, which can make the trunk and legs move forward, backward and laterally independently. Since the hip is the center of a series of body movements, you are more likely to get injuries in your daily life (1, 2). Hip fracture is common in older people over 60 years old, who are often accompanied by osteoporosis and are prone to hip fracture when suffering from low energy trauma (3, 4). Hip replacement is a surgical method for the treatment of hip diseases such as hip fracture in the elderly, which can effectively correct hip deformity, relieve hip pain, help patients recover hip function, improve the quality of life, and significantly improve the clinical symptoms and clinical manifestations of patients (5, 6). The choice of anesthesia in clinical practice is determined by different factors, such as patient differences, pre-operative complications, risk of post-operative complications, and doctors’ clinical experience (7, 8). As there are many chronic basic diseases in the elderly patients due to pathological and physiological changes, anesthesia has a greater impact on the postoperative prognosis of elderly patients, so the choice of anesthesia brings challenges to clinical work (9, 10). Intraspinal anesthesia is one of the current anesthesia methods for hip fracture, and it is commonly used in China. However, when the anesthesia level of elderly patients is too high, it can lead to severe hypotension, or even affect respiratory function. The application of intraspinal anesthesia to hip fractures in the elderly is limited by the fact that degenerative lumbar intervertebral stenosis and ligament calcification can lead to difficulty or failure in puncture in the elderly (11, 12). Dexmedetomidine is a highly selective *α*2- adrenoceptor agonist, which has sedative, analgesic,, anti-anxiety, inhibition of sympathetic nerve activity, and stability of hemodynamics. Studies have found that dexmedetomidine can enhance the effect of propofol and opioids, stabilize cerebral blood flow, and has a neuroprotective function (13–15). The purpose of this study was to investigate the anesthesia effects of dexmedetomidine combined with intraspinal anesthesia in hip joint replacement and analyze its effects on postoperative stress response, the incidence of delirium, immune function and inflammation indexes of patients.

## Materials and Methods

### Patients

A total of 42 patients who underwent hip replacement in our hospital from March 2020 to June 2021 were selected as the research subjects. Inclusion criteria: age ≥60 years old; All patients underwent hip replacement; All patients met the standards of spinal anesthesia; American Society of Anesthesiologists (ASA) grades II to III; Patients with normal cardiopulmonary function. Exclusion criteria: With coagulation disorders; Patients with hematopoietic system diseases; Patients with arrhythmia and severe conduction block; Patients with history of delirium and dementia before operation. All the patients were randomly divided into a control group and an observation group, 21 cases in each group. There was no significant difference in general information between the two groups (*P* > 0.05). As shown in Table 1.

**Table 1:** Comparison of general data of patients between the two groups.

Group	Gender		Age (years)	BMI (kg/m^2^)	ASA		Disease site		Course of disease (years)
	Male	Female			II	III	Left	Right	
Control group	28	21	69.53 ± 5.16	23.46 ± 2.19	16	5	11	10	6.08 ± 2.04
Observation group	30	19	69.68 ± 5.29	23.59 ± 2.24	14	7	9	12	5.96 ± 2,15
t/*χ*^2^	0.383		0.142	0.291	0.467		0.382		0.186			
*P*	0.536		0.887	0.772	0.495		0.537		0.854			

### Treatment Methods

After preoperative preparation and exclusion of surgical contraindications, the patient was sent to the operating room for hip replacement. Routine intravenous channels were opened before surgery, blood pressure, heart rate, pulse, blood oxygen saturation and Electroencephalogram (EEG) dual frequency index were continuously detected, and oxygen inhalation by mask was continued during surgery. During the operation, oxygen was continuously inhaled through face mask. In the control group, intraspinal anesthesia was adopted. The patient was in the lateral decubitus position, and epidural puncture was performed using the intervertebral space of L2 and L3 as the puncture points. After successful puncture, a tube was inserted into the epidural space with the catheter pointing towards the head end. The spinal anesthesia was performed with 1 ml of 0.5% bupivacaine (Shandong Hualu Pharmaceutical Co., LTD., National Drug Approval: H37022107), and the anesthesia plane was controlled below the 10th thoracic vertebra (T10) plane. On the basis of the control group, patients in the observation group were additionally sedated with dexmedetomidine: The method of intraspinal anesthesia was the same as above. After the spinal canal was fixed with the anesthesia plane, dexmedetomidine (Jiangsu Nhwa Pharmaceutical Co., Ltd., National Drug Approval Code: H20110085) hydrochloride injection at 1 μg/kg was given, and the infusion pump was connected after intravenous infusion for 10–15 min, for continuous intravenous pumping at the rate of 0.2 μg/(kg·h).

Both groups were given automatic intravenous analgesia to relieve postoperative pain. The analgesic formula was 0.9% sodium chloride injection 100 ml and 2 μg/kg sufentanil. If the patient has severe postoperative pain, 40 mg parecoxib can be injected intravenously to keep the patient's postoperative pain visual analog score below 4 points.

### Observation Indicators

#### Anesthesia Effect index

The onset time and maintenance time of sensory and motor nerve block in the two groups were recorded.

#### Stress Response Indicators

The levels of serum cortisol (Cor), blood glucose (Glu), epinephrine (E) and norepinephrine (NE) in the two groups were measured preoperatively and 1d after operation using an automatic biochemical analyzer (Mindray Automatic Biochemical analyzer BS-280).

#### Occurrence of Delirium

3 days after operation, the incidence of postoperative delirium in the two groups was recorded, and the incidence of delirium was calculated. Incidence of delirium = cases of delirium/total cases  × 100%.

#### Immune Function Indicators

The levels of T lymphocyte subsets (CD3+, CD4+, CD8+, CD4+/CD8+) were measured by FACSCount flow cytometry produced by BD Company in the United States and supporting reagents.

#### Inflammatory Indicators

The levels of tumor necrosis factor-α (TNF-α) and interleukin-6 (IL-6) in serum of both groups were detected by ELISA before and 1d after surgery. The above kits were purchased from Shanghai Enzyme-linked Biotechnology Co., LTD.

#### Adverse Reactions

Postoperative observation was made for nausea, vomiting, dizziness and other adverse reactions in both groups.

### Statistical Methods

All data were processed with SPSS 22.0 statistical software. The enumeration data were examined by X^2^ test and expressed by [n(%)], the measurement data were examined by t-test and expressed by ($\bar{x}\pm s$). The difference is statistically significant when *P* < 0.05.

## Results

### Comparison of Anesthesia Effect Indexes Between the Two Groups

**Figure 1:**
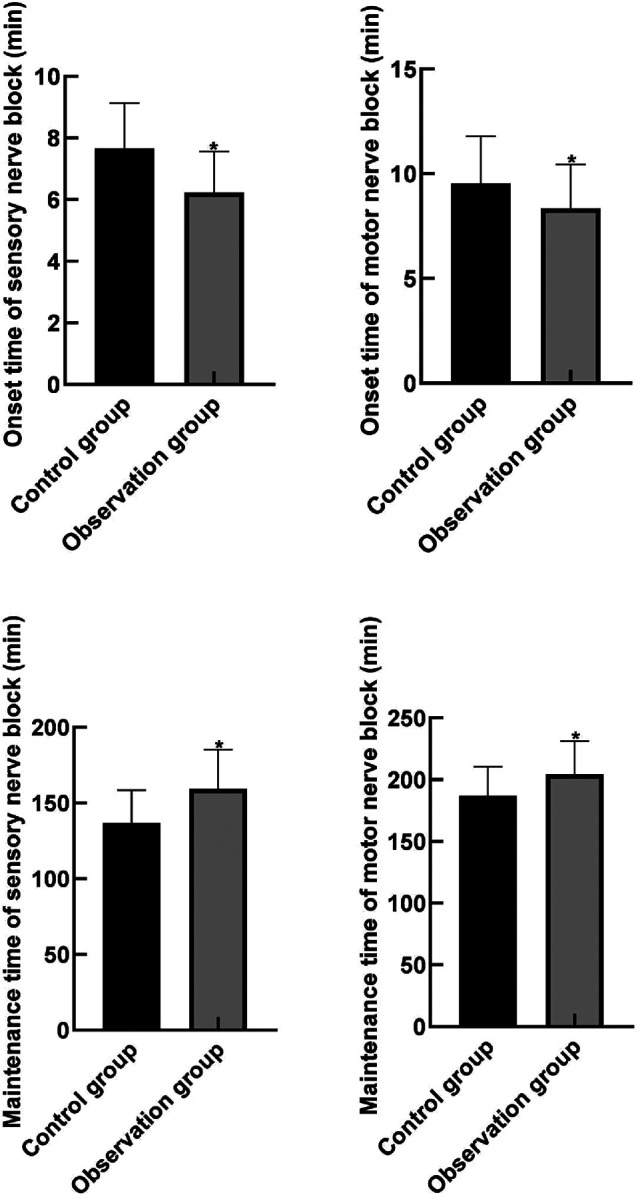
Comparison of anesthesia effect indexes between the two groups. Note: Compared with the control group, *P < 0.05.

The onset time of sensory and motor block in the observation group was lower than that in the control group, and the retention time of sensory and motor block was higher than that in the control group (*P* < 0.05). As shown in Figure 1.

### Comparison of Stress Response Indicators Between the Two Groups

**Figure 2:**
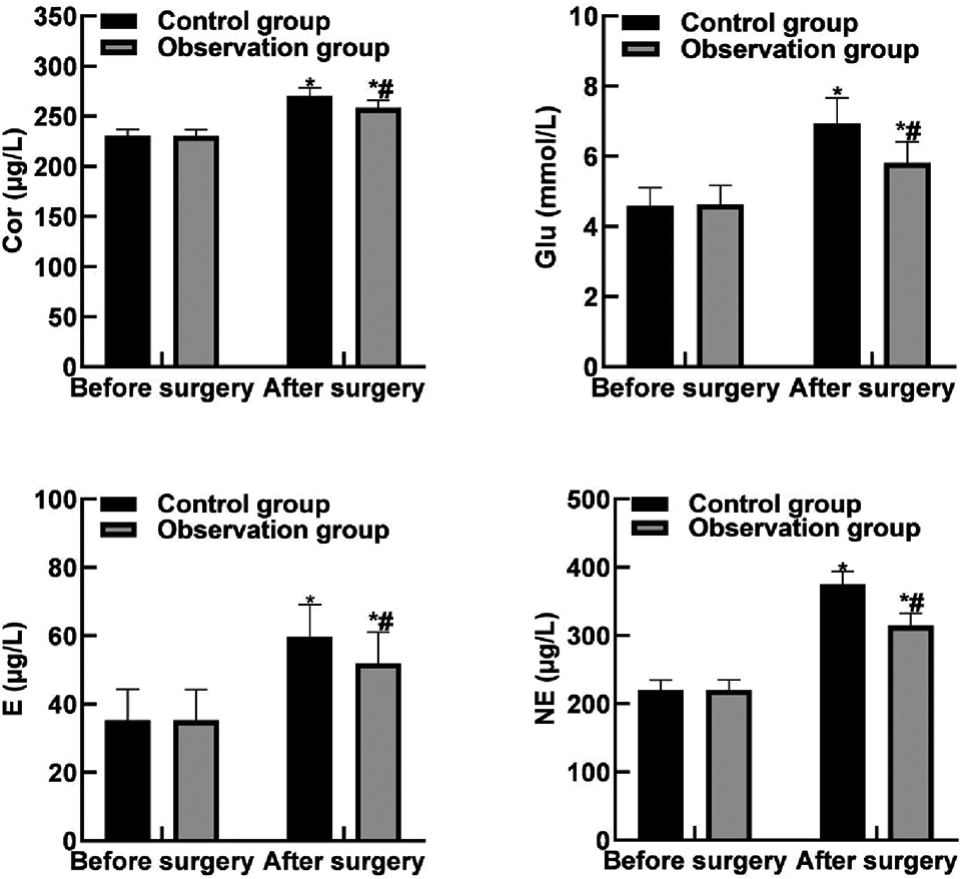
Comparison of stress response indicators between the two groups**.** Note: Compared with before surgery, *P < 0.05. Compared with the control group, ^#^*P* < 0.05.

After surgery, the levels of Cor, Glu, E and NE in the two groups were higher than those before surgery, and the levels of Cor, Glu, E and NE in the observation group were lower than those in the control group (*P* < 0.05). As shown in Figure 2.

### Comparison of Delirium Occurrence Between the Two Groups

After surgery, the incidence of delirium in the control group and the observation group was 28.57% (6/21) and 4.79% (1/21), respectively. The incidence of postoperative delirium in the observation group was lower than that in the control group (*P* < 0.05).

### Comparison of Immune Function Indicators Between the Two Groups

**Figure 3:**
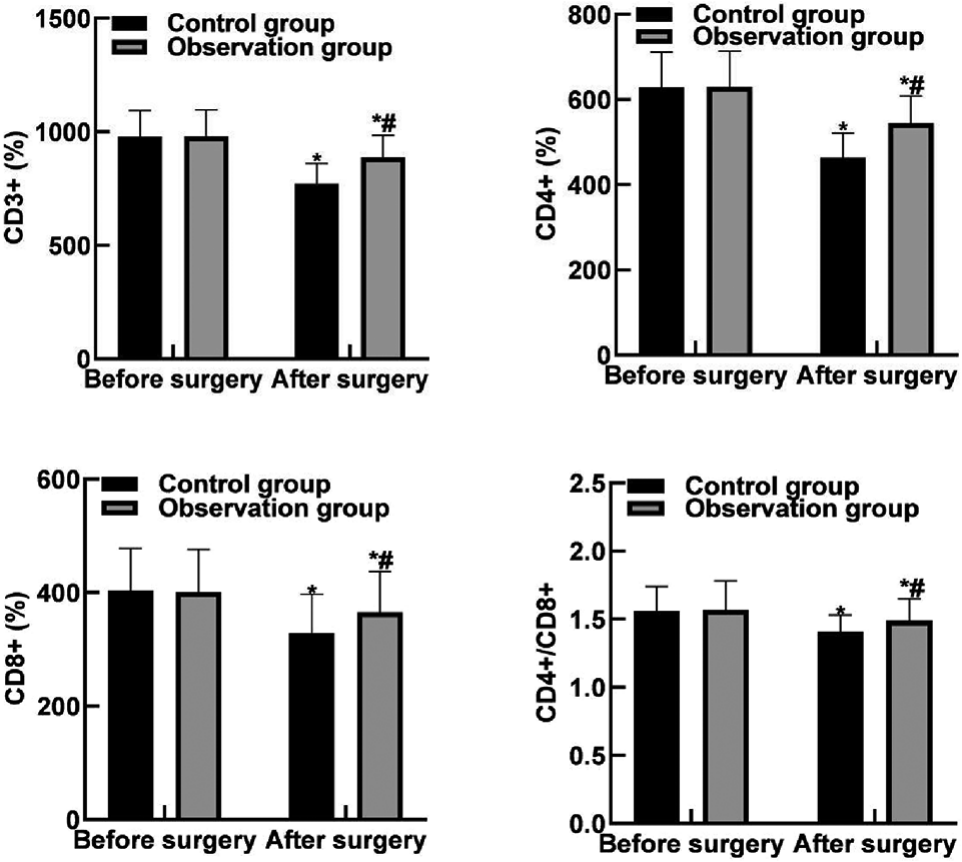
Comparison of immune function indicators between the two groups**.** Note: Compared with before surgery, *P < 0.05. Compared with the control group, ^#^*P* < 0.05.

After surgery, the levels of CD3+, CD4+, CD8+, and CD4+/CD8 + in the two groups were lower than those before surgery, and the levels of CD3+, CD4+, CD8+, and CD4+/CD8 + in the observation group were higher than those in the control group (*P* < 0.05). As shown in Figure 3.

### Comparison of Inflammatory Indexes Between the Two Groups

**Figure 4:**
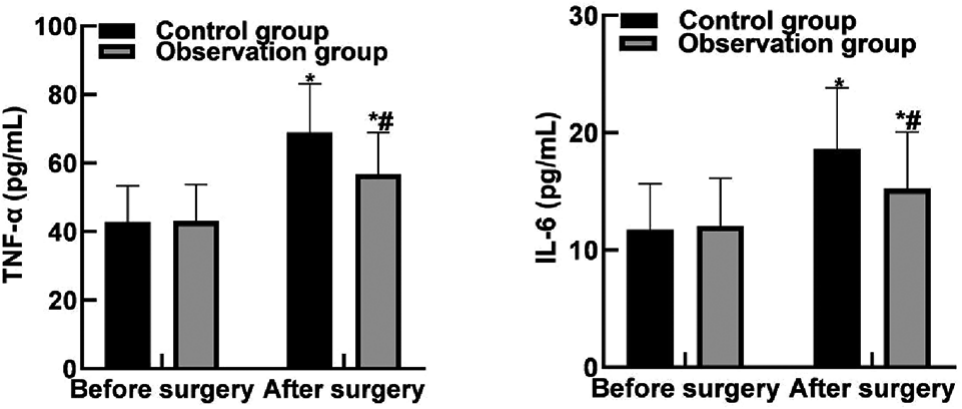
Comparison of inflammatory indexes between the two groups**.** Note: Compared with before surgery, *P < 0.05. Compared with the control group, ^#^*P* < 0.05.

After operation, the levels of TNF-α and IL-6 in both groups were higher than those before operation, and the levels of TNF-α and IL-6 in observation group were lower than those in control group (*P* < 0.05). As shown in Figure 4.

### Adverse Reactions of the Two Groups

After operation, there were 2 cases of nausea, 1 case of vomiting, 1 case of dizziness and 1 case of hypotension in the control group, and the total incidence of adverse reactions was 23.81% (5/21). In the observation group, there was 1 case of nausea, 1 case of dizziness and 1 case of hypotension, and the total incidence of adverse reactions was 14.29 (3/21). The incidence of adr in observation group was slightly lower than that in control group, but the difference was not statistically significant (*P* > 0.05). As shown in Table 2.

**Table 2:** Adverse reactions of the two groups.

Group	Nausea	Vomiting	Dizziness	Hypotension	Total incidence
Control group	2	1	1	1	5 (23.81)
Observation group	1	0	1	1	3 (14.29%)
χ^2^					0.618
*P*					0.432

## Discussion

Elderly patients undergoing hip replacement are prone to stress reaction during the operation because they often suffer from various basic diseases of the system, such as diabetes and hypertension, and the decline of body organ function, resulting in low compensatory function of the cardiovascular system and reduced regulation ability of the circulatory system. In addition, due to slow drug metabolism, patients are prone to delayed awakening or even temporary brain dysfunction after surgery, which increases the risk of complications and affects the patient's rehabilitation (16, 17). Previous studies have found that intraspinal anesthesia has the characteristics of good analgesic effect, small drug consumption, and the block range is limited to one side, which can effectively reduce the sympathetic activity and reduce vasodilation, thereby reducing the damage to the circulatory system and the occurrence of adverse events, and effectively meeting the basic needs of hip replacement in the elderly (18, 19). Our patient avoided endotracheal intubation and mechanical ventilation with relatively low respiratory complications; The incidence of nausea and vomiting is low; Epidural analgesia can be performed with good postoperative analgesic effect. However, for the routine use of anticoagulants during perioperative period, the use of epidural analgesia is limited to a certain extent due to the risk (20, 21). In addition, the change of body position during anesthesia may result in the displacement of the fracture end, causing damages to the peripheral nerves and blood vessels, or even aggravation. Moreover, patients under simple intraspinal anesthesia are in a awake state and easily suffer from tension, anxiety and other bad emotions. With the use of traditional sedative auxiliary drugs, elderly patients are often more sensitive, which finally leads to different degrees of inhibition of respiratory cycle, thus affecting the safety during the operation (22, 23). Therefore, effective sedation is still need to stabilize that patient's vital signs.

The results of this study showed that the onset time of sensory and motor block in the observation group was lower than that in the control group, and the retention time of sensory and motor block was higher than that in the control group. It indicated that dexmedetomidine combined with intraspinal anesthesia had better anesthesia effect in hip joint replacement. The reason for this analysis is that dexmedetomidine has a high selectivity for *α*2- adrenergic receptor agonists, and therefore it works more quickly and is less toxic. Dexmedetomidine can enhance the anesthetic effect in the spinal canal by inhibiting the central and peripheral sympathetic nerves, and can also block the release of neurotransmitters by acting on presynaptic C fibers and spinal motor neurons (24, 25).

Stress response is a series of physiological and psychological changes generated by the human body in response to external stimuli. Moderate stress response is conducive to the body to adapt to the external stimuli, while excessive stress response can cause various system dysfunction, or even life-threatening (26, 27). Due to the degradation of body function, the sensitivity of various systems of the body to external stimuli is decreased in elderly patients, and they are more likely to have stress responses. Surgical trauma and pain are the stress source of elderly patients with hip replacement. If the stress response of patients is not effectively controlled, it is likely to induce cardiovascular events and other accidents, increasing the risk of surgery and anesthesia. Cor is an adrenocortical hormone that can effectively regulate the relationship between immune cells and inflammation, blood vessels and blood pressure, and its abnormal expression is often closely related to stress response. Glu is an important source of energy. Under stress state, the excitability of sympathetic nervous system will be enhanced, and the secretion of glucocorticoids and catecholamine will be increased, resulting in the decrease of glucagon and insulin contents, as well as the initiation of hepatic glycogen decomposition and gluconeogenesis, further resulting in the elevation of blood glucose. E mainly comes from adrenal gland, and can cause increased heart rate, increased cardiac output, and enhanced myocardial contractility, with the increased content suggesting that the body was stimulated by injuries. NE is not only a neurotransmitter but also a hormone, which can cause the contraction of venules and arterioles by acting on *α* receptor, and increase the peripheral resistance, leading to an increase in blood pressure and slow heart rate. The serum content of NE is positively correlated with the degree of stress response (12, 28). The results of this study showed that after surgery, the levels of Cor, Glu, E and NE in the observation group were lower than those in the control group. These results indicated that dexmedetomidine combined with intraspinal anesthesia could reduce the stress response of surgery and the change amplitude of stress hormones after surgery. The reason for this was analyzed as follows: Dexmedetomidine can directly act on the posterior horn of gray matter in the spinal cord of patients, thus triggering the hyperpolarization of postsynaptic membrane and inhibiting the convergence of noxious mediators, thus greatly reducing the pain of patients and relieving stress response of the body.

Delirium is a cognitive or sensory disorder associated with inattention that develops over a short period of time and fluctuates over time. Postoperative delirium is a heterogeneous disorder characterized by dramatic changes in mental status, such as changes in attention and consciousness (29, 30). Postoperative delirium has a high incidence in patients undergoing hip replacement, especially in patients with hip fractures, which is as high as 35% to 65%. The incidence of delirium is generally high because preoperative fractures (acute trauma), pain, chronic inflammation and subsequent surgical treatment can all be contributing factors to systemic reactions, and because hip replacements are generally performed in older patients (31, 32). The results of this study showed that the incidence of postoperative delirium in the observation group was lower than that in the control group. These results indicated that dexmedetomidine combined with intraspinal anesthesia could reduce the incidence of postoperative delirium in patients. The reason was analyzed as follows: The sleep state induced by dexmedetomidine was natural non-oculomotor sleep, which could effectively promote the recovery and repair of nervous and immune system of elderly patients, avoid the impairment of cognitive function and physiological function, thus shorten the awakening time and reduce the incidence of postoperative delirium. Dexmedetomidine combined with intraspinal anesthesia can produce a synergistic effect, which is conducive to reducing the stress source, reducing the stress state of patients, and improving the safety of surgery.

T-lymphocyte level can reflect the changes of immune function in patients and directly affect complications, rehabilitation and prognosis of patients. CD3+, CD4+, CD8+, and CD4+/CD8+ are common cellular immune indicators (33, 34). CD3+ mainly exists on the cell surface and is involved in T cell activation signal transduction, thus initiating the immune response. CD4+ can assist the expression of T cells, which can guide the body's resistance to inflammatory cells, so as to enhance the body's anti-toxicity ability. The results of this study showed that after surgery, the levels of CD3+, CD4+, CD8+, and CD4+/CD8+ in the observation group were higher than those in the control group. These results indicated that dexmedetomidine combined with intraspinal anesthesia could improve the postoperative immune function of patients and inhibit the expression of T lymphocyte subsets. The results of this study showed that the levels of TNF-α and IL-6 in the observation group were lower than those in the control group. These results indicate that dexmedetomidine combined with spinal anesthesia can effectively reduce the inflammatory response level caused by surgical trauma. The reason is analyzed that dexmedetomidine can regulate the release of inflammatory factors by macrophages and monocytes, exert anti-inflammatory effect by reducing the chemotaxis of inflammatory cells and enhancing cell-mediated immune response, and inhibit the TOLL-like receptor 4 inflammatory pathway, thereby exerting anti-inflammatory effect.

## Conclusion

Dexmedetomidine combined with intraspinal anesthesia has good anesthesia effect in hip joint replacement, which can greatly reduce the stress response of patients, reduce the incidence of postoperative delirium of patients, and effectively restore the immune function of patients, reduce the level of inflammatory response, and has high clinical application value.

## Data Availability

The original contributions presented in the study are included in the article/Supplementary Material, further inquiries can be directed to the corresponding author/s.
